# Knowledge, Attitude and Practice on Antibiotics Use and Its Resistance among Medical Students in a Tertiary Care Hospital

**DOI:** 10.31729/jnma.4224

**Published:** 2019-04-30

**Authors:** Ruchi Shrestha

**Affiliations:** 1 Department of Pharmacology, Kathmandu University School of Medical Sciences, Dhulikhel, Nepal

**Keywords:** *antibiotics*, *attitude*, *knowledge*, *medical students*

## Abstract

**Introduction:**

Antimicrobial resistance is an urgent and serious global health problem, demanding considerable attention from health care professionals worldwide. The unavoidable consequence of widespread use of these agents has been the reason for emergence of antibiotic resistant pathogens, leading to increase in need for new drugs. This study aims to access knowledge, attitude and practice towards antibiotics use and its resistance in undergraduate medical students.

**Methods:**

A descriptive cross-sectional study was conducted among 228 undergraduate medical students studying in a tertiary care hospital in February, 2018. Data was collected through self administered questionnaire and was entered in Statistical Package for Social Sciences. Descriptive statistical analysis was carried out to find out knowledge, attitude and practice on antibiotics use and its resistance on medical students.

**Results:**

The mean knowledge, attitude and practice score towards antibiotics use among students was 7.44±1.26, 3.35±1.12 and 5.06±1.45 respectively. Out of total students, only 39 (17.1%) had good knowledge and practice whereas 114 (50%) had good attitude towards antibiotics use. Approximately, all 224 (98.2%) students were aware that antibiotics are useful for bacterial infection. Fifty two (22.8%) students said that antibiotics are safe drugs, therefore, can be used commonly.

**Conclusions:**

Although half of the students had good attitude, majority had moderate knowledge and practice towards antibiotics use. Adequate educational training should be provided to future doctors regarding proper prescribing, dispensing and usage of antibiotics.

## INTRODUCTION

Antibiotics have not only saved patient's lives, they have also played important role in achieving major advances in therapeutics.^1^ However, the emergence of bacterial strains resistant to antimicrobial agents presents a growing concern worldwide and has lead to global antibiotics crisis.^2^

Medical students are the future doctors. To promote judicious use of antibiotics, adequate training should be provided to undergraduate medical students regarding proper prescribing, dispensing and use of antibiotics.^3^ Importance of these issues should be emphasized at this crucial time, because once they become qualified, it is difficult to change their deeply rooted views and behavior.^4^

This study was undertaken among first and second year undergraduate medical students to assess their Knowledge, Attitude and Practices (KAP) regarding antibiotics use and its resistance.

## METHODS

This is descriptive cross-sectional study conducted in Universal College of Medical Sciences (UCMS), Bhairahawa, Nepal. Data collection was done from 6^th^ to 22^nd^ February, 2018. Ethical approval was obtained from the Institutional Review Committee of UCMS (UCMS/IRC/012/18). All MBBS and BDS students studying in first and second year were included in the study. Consent was taken from each student prior to data collection.

Semi-structured questionnaire were developed after reviewing previously published related studies with few modifications.^6–8^ Questionnaire was divided into 4 parts. First part included demographic profile of students, 2^nd^ part consisted of nine questions to assess the knowledge of students about antibiotics and its resistance, 3^rd^ part included five questions to access the attitude of students towards rational use of antibiotics and 4^th^ part consisted of eight questions which was related to practices to self medication of antibiotics. Knowledge and attitudes regarding antibiotic and its resistance were analysed using 5-point Likert scale, whose responses ranged from 1=strongly agree, 2=agree, 3=neither agree nor disagree, 4=disagree and 5=strongly disagree. Student's self reported practices regarding antibiotic usage was also assessed using Likert scale whose responses ranged from ‘always’ to ‘never’. The questionnaire was distributed to first and second year MBBS and BDS students during one of their pharmacology practical classes. Each participant was allotted 20 minutes to answer the questionnaire in the form of options which he/she felt appropriate to answer.

One point was given for each correct answer whereas no deduction was done for wrong, neither agree nor disagree and sometimes responses. In knowledge questionnaire on antibiotics use, those who got score between 7–9, 4–6 and 0–3 were considered as having good, moderate and poor knowledge respectively. In attitude questionnaire, those who got score between 4–5, 2–3 and 0–1 were considered as having good, moderate and poor attitude respectively. In practice questionnaire, those who got score between 7–8, 4–6 and 0–3 were considered as having good, moderate and poor practice respectively on antibiotics use.

The collected data was coded and entered in Statistical Package for Social Sciences (SPSS) version 16. Frequency and percentage were calculated to find out the students KAP towards antibiotics use and its resistance.

## RESULTS

In this study, questionnaire was distributed to all the students i.e. 290 out of which, there were 252 returns. Twenty four questionnaires were subsequently excluded because of incomplete data. The final response rate was 228 (78.62%), out of which 124 (54.4%) were male and 104 (45.6%) were female. The mean age of study participants was found to be 19.83 ± 1.43 years.

To make the analysis easier in knowledge and attitude questionnaire, “Strongly Agree” and “Agree” was merged into “Agree”, and “Strongly Disagree” and “Disagree” was merged into “Disagree”. Majority 211 (92.5%) of the students were aware of the fact that indiscriminate and injudicious use of antibiotics can lead to ineffective treatment. Antibiotics were first choice drugs for 70 (30.7%) students when they had cough and sore throat. More than half 120 (52.6%) students never discarded remaining, leftover antibiotics (Table 1, 2 and 3).

**Table 1:** Knowledge on antibiotics use and its resistance.

Questions	Agree n (%)	Neither agree nor disagree n (%)	Disagree n (%)
Antibiotics are useful for bacterial infection	224 (98.2)	0 (0)	4 (1.8)
Antibiotics are useful for viral infection	59 (25.9)	11 (4.8)	158 (69.3)
Use of antibiotics will speed up recovery from flu and cold	105 (46.1)	13 (5.7)	110 (48.2)
Antibiotics resistance means that if antibiotics are taken too often, they are less likely to work in future	190 (83.3)	3 (1.3)	35 (15.4)
Antibiotics resistance is important and serious global public health issue	202 (88.6)	15 (6.6)	11 (4.8)
Indiscriminate and injudicious antibiotics use can lead to			
i) Ineffective treatment	211 (92.5)	5 (2.2)	12 (5.3)
ii) Increased adverse effects	212 (93)	7 (3.1)	9 (3.9)
iii) Increased cost burden to the patient	203 (89.1)	11 (4.8)	14 (6.1)
Antibiotics can cause secondary infections after killing good bacteria present in the body	168 (73.7)	24 (10.5)	36 (15.8)

**Table 2:** Attitude towards antibiotics use.

Questions	Agree n (%)	Neither agree nor disagree n (%)	Disagree n (%)
Antibiotics are safe drugs, hence they can be used commonly	52 (22.8)	30 (13.2)	146 (64)
Skipping one or two doses does not contribute to development of antibiotics resistance	59 (25.8)	12 (5.3)	157 (68.9)
Adverse effects of antibiotics are reduced by using more than one antibiotic at a time	66 (29))	29 (12.7)	133 (58.3)
Injudicious use of antibiotics shortens the duration of illness	30 (13.2)	18 (7.9)	180 (78.9)
When you have a cough and sore throat, antibiotics are the first drug of choice for early treatment and to prevent emergence of resistant strains	70 (30.7)	15 (6.6)	143 (62.7)

**Table 3:** Self reported practices on antibiotics.

Questions	Always n (%)	Sometimes n (%)	Never n (%)
The doctor prescribes a course of antibiotic for you. After taking 2–3 doses you start feeling better			
i) You stop taking the further treatment?	5 (2.2)	52 (22.8)	171 (75)
ii) Do you save the remaining antibiotics for the next time you get sick?	13 (5.7)	53 (23.2)	162 (71.1)
iii) Do you discard the remaining, leftover medication?	28 (12.3)	80 (35.1)	120 (52.6)
iv) Do you give the leftover antibiotics to your friend/family members if they get sick?	13 (5.7)	85 (37.3)	130 (57)
v) Do you complete the full course of treatment?	180 (78.9)	41 (18)	7 (3.1)
Do you consult doctor before starting an antibiotic?	190 (83.3)	37 (16.2)	1 (0.4)
Do you check expiry date of the antibiotic before using it?	223 (97.8)	4 (1.8)	1 (0.4)
Do you prefer to take antibiotic when you have cough and sore throat?	15 (6.6)	147 (64.5)	66 (28.9)

The mean KAP score among students in this study was 7.44±1.26, 3.35±1.12 and 5.06±1.45 respectively. Out of total students, 39 (17.1%) had good knowledge and practice whereas 114 (50%) had good attitude towards antibiotics use (Table 4 and 5).

**Table 4:** KAP score.

	Minimum score	Maximum score	Mean score
Knowledge	1	9	7.44±1.26
Attitude	1	5	3.35±1.12
Practice	1	8	5.06±1.45

**Table 5:** KAP level of students

	Good n (%)	Moderate n (%)	Poor n (%)
Knowledge	39 (17.1)	153 (67.1)	36 (15.8)
Attitude	114 (50)	101 (44.3)	13 (5.7)
Practice	39 (17.1)	151 (66.2)	38 (16.7)

## DISCUSSION

Antibiotics are the most frequently prescribed drugs, but often misused.^9^ Antibiotic resistance is increasing all over the world and presents a growing concern worldwide.^10^ The clinical effectiveness of antibiotics depends on their correct use by patients, physicians and retailers.^11^ Prescriber's decisions may be influenced by factors such as fear of losing their patients, lack of information on rational use of antibiotics, excessive and unnecessary antibiotic prescribing, incorrect dosage or route of administration, antibiotic prescribing for non-bacterial infections, patient demands and self prescribing.^12–15^ Patient factors relating to incorrect antibiotic use include self-medication, sharing medication, not taking full course of treatment and keeping part of the course for next time.^16^ Poor infection control practices and increasing use of antibiotics in agriculture, farming and aquaculture has also led to global concern for antibiotics resistance.^1,17^

Majority 88.6% of the students in this study were well aware of the global problem of antimicrobial resistance. The finding was similar to the study done in Congo in medical students and practicing medical doctors in which 85.4% participants said antibiotics resistance is important global problem.^18^ Approximately 98% students in our study were aware of the fact that antibiotics are useful for bacterial infection which was similar to the study done in Italy which suggested 95.2% medical students were aware that antibiotics are useful for treatment of bacterial infections.^6^ However, in this study, 25.9% students agreed that antibiotics are also useful for viral infections and 46.1% said use of antibiotics causes speed recovery from cold and flu. The findings were contrast to the study done in Iran^19^ which reported 100% of the medical students believed that antibiotics do not cure viral illnesses and in China^7^ in which 72.6% students said use of antibiotics speed up recovery from flu and cold. Such wrong beliefs may lead to inappropriate increased rates of antibiotic consumption, which can result in increase in bacterial resistance.^20^

In response to survey question, “antibiotics are safe drugs; hence they can be used commonly”, 64% disagreed. The response was similar to Indian study in which 78.4% students disagreed to the same question.^8^ Approximately 30% students said that antibiotics are the first choice drugs when they get cough and sore throat whereas 52.32% gave the same response in a study done in India.^21^

In response to self medication questions related to antibiotics, 83.3% students said they always consulted doctors before taking antibiotics, which was similar to the study carried out in Italy which reported 81.38% medical students consulted doctors before taking antibiotics.^6^ However, study done in general public revealed that approximately one-third had got antibiotics directly from pharmacy without physician consultation as shown by the study done in Italy (32.7%),^22^ and Jordan (30.5%).^23^ Majority 78.9% of the students completed the full course of antibiotics which was similar to the study in which 74.2%^8^ and 94.18%^20^ medical students completed the course.

Existing gaps are present in the knowledge, attitude and practice of antibiotics use in medical and non medical students as shown by various studies. Overall knowledge, attitude and perception of medical students were better when compared to non medical students whereas behaviour towards antibiotics use was poor in medical students when compared to non medical students as shown by the study in China.^7^ In study done in Libya where rate of self medication was compared, 43% of the medical students were self medicated as compared to 31% of non medical students.^24^ This could be due to their self confidence and knowledge gained from medical study.^25^

The present study had several limitations. Opinions of students of single teaching hospital were taken. So, the results cannot generalize the whole population of medical students. In order to know the exact scenario, perception of all the medical students in the national level should be included. Secondly, all the answers were not given honestly as some of the students discussed among them while filling the questionnaire. Finally, the number and way of questions may not reflect the real KAP of the students.

## CONCLUSIONS

This study revealed an important insight regarding the KAP regarding antibiotics use and its resistance among medical students. Although half of the students had good attitude, majority had moderate knowledge and practice towards antibiotics use. Medical students are future doctors who are going to practice in medical field, so if they have clear concept about antibiotics like when to use and when not, antibiotics resistance can be reduced to greater extent. The medical education strategies should aim to provide adequate training on the rational use of antibiotics and not only to increase the knowledge but also to change the behaviour and practices among medical students regarding antibiotics use. Effective undergraduate curriculum should be developed to enhance future doctor's knowledge, attitude and practices for usage of antibiotics.
